# Half metal phase in the zigzag phosphorene nanoribbon

**DOI:** 10.1038/s41598-018-21294-0

**Published:** 2018-02-13

**Authors:** Yi Ren, Fang Cheng, Z. H. Zhang, Guanghui Zhou

**Affiliations:** 1 Department of Physics and Electronic Science, Changsha University of Science and Technology, Changsha, 410004 China; 2 Department of Physics and Key Laboratory for Low-Dimensional Quantum Structures and Manipulation (Ministry of Education), and Synergetic Innovation Center for Quantum Effects and Applications of Hunan, Hunan Normal University, Changsha, 410081 China

## Abstract

Exploring half-metallic nanostructures is a crucial solution for developing high-performance spintronic devices. Black phosphorene is an emerging two-dimensional material possessing strong anisotropic band structure and high mobility. Based on the first principles calculations, we investigated the electronic and magnetic properties of zigzag phosphorene nanoribbons (ZPNRs) with three different functionalization groups (OH/CN, OH/NO_2_, NH_2_/NO_2_) at the edges. We find that the interplay between edge functionalization and edge oxidation can induce the half metal phase in the ZPNRs, and the half metal phase can be controlled by the external transverse in-plane electric field and the proportion of the functional groups and edge oxidation. The results may pave a new way to construst nanoscale spintronic devices based on black phosphorene nanoribbons.

## Introduction

Phosphorene, the single- or few-layer form of black phosphorous, has attracted intensive attention because of its unique electronic properties and potential applications in nanoelectronics^1–9^. Comparing with gapless graphene with linear chiral isotropic energy dispersion, black phosphorene (BP) possesses thickness-dependent bandgap and strongly anisotropic band structures. Few layer and eventually monolayer BP possesses a direct band gap at Γ point ranging from 0.3 eV to 2 eV^10^. To date, various interesting properties for BP have been predicted, particular those related to strain induced gap modification^6^, large tunable optical properties^11–13^, one-dimensional (1D)-like excitons^14^. Further, the field-effect-transistor (FET) based on few layer black phosphorus is found to have an on/off ratio of 10^5^ and a carrier mobility at room temperature as high as 10^3^ cm^2^/V⋅s^1,15^. Atomic layer deposited AlO_*x*_ overlayers effectively suppress ambient degradation, allowing encapsulated BP FETs to maintain high on/off ratios of ~10^3^ and mobilities of ~100 cm^2^ V^−1^s^−1^ for over two weeks in ambient conditions^16^. Monolayer BP has also been implemented into various electronic device applications including gas sensor^17^, solar cell application^18^ due to its sizable band gap as compared to graphene and because of its higher carrier mobility as compared to MoS_2_.

Due to the unique anisotropic band structure of BP, the electronic structures of the BP nanoribbons depend sensitively on the crystal orientation of the ribbons. Two typical crystal orientations were generally explored, namely the zigzag-phosphorene nanoribbons (ZPNRs) and the armchair-phosphorene nanoribbons (APNRs). It is demonstrated that the controlled structural modification of few-layer BP along arbitrary crystal directions with sub-nanometer precision for the formation of few-nanometer-wide armchair and zigzag PNRs^19^. Theoretical studies pointed out that compared with APNRs, the ZPNRs possesses a stronger quantum size effect^20,21^ and edge states^22^. Hydrogen or halogen passivation of ZPNRs displays nonmagnetic semiconducting behaviors, while chalcogen- and oxygen-passivated ZPNRs exhibit magnetic phases^23,24^. The magnetic properties of ZPNRs passivated by iron group atoms are also studied^25^. Different edge functionalization groups would have a significant impact on the electronic structure of the ZPNRs near the Fermi level.

Half metallicity is one of the central issues in spintronics field^26,27^. Half metallicity occurs when one of the electron spin orientation shows insulating behavior while the other shows metallic behavior^28^. Theoretical calculations have shown that half-metallicity may be realized in the zigzag graphene nanoribbon(ZGNR) either by applying a high in-plane homogeneous electric field or by chemically functionalizing zigzag-edges of the graphene nanoribbon with different groups such as H, COOH, OH, NO_2_, NH_3_, CH_3_, etc^29–32^. First-principles calculations within the local spin-density approximation reveal half metallicity in zigzag boron nitride nanoribbons^29,33^ and graphene/boron nitride lateral supperlattices^34^.

In this paper, we investigated theoretically the effect of edge configurations on the electronic structure of a reconstructed zigzag edge of PNRs and found a general condition to induce a half-metallicity state. The ZPNRs with functionalization group OH/NO_2_(NH_2_/NO_2_) and oxidation at the edges are spin-polarized semimetals in the absence of an external electric field. In contrast to graphene nanoribbon^29^, our proposal does not require external electric fields. The inherent electric field induced by the asymmetric functional saturation makes the charge transfer between two edges, that is, the electron accumulation/depletion at both egdes. The unsaturated bonds bring magnetic moments in oxygen-saturated ZPNRs. Different oxygen ratios lead to different total magnetic moments of the ZPNRs with edge functionalization groups and edge oxidation. Therefore, this half metal phase can be tuned by external electric fields and the edge functionalization groups. These unique properties make the ZPNRs an attractive candidate for flexible nanoscale spintronic devices.

## Structure and Methods

The geometric optimizations as well as calculations of the electronic structures for the ZPNRs are performed by first-principles method based on the density functional theory (DFT) implemented in the Atomistix ToolKit (ATK) as well^35–37^. We employ Troullier-Martins norm-conserving pseudopotentials to represent the atom core and linear combinations of local atomic orbitals to expand the valence states of electrons. The spin-dependent generalized gradient approximation (SGGA) is used as the exchange–correlation functional. Considering the influence of the atom polarization, the wave function is expanded by double-zeta plus polarization (DZP) basis for all atoms. The k-point sampling is 1 × 1 × 100 Å in the *x*, *y*, and *z* directions, respectively, where *z* is the period direction of nanoribbon, and the cut off energy is set to 150 Ry. For models studied, a 15 Å vacuum slab is used to eliminate interaction between the model and its “ images”, and all the geometries are optimized until all residual forces on each atom are smaller than 0.01 eV/*Å*. Our calculated lattice constants for bulk black phosphorus are *a* = 3.3136 Å, *b* = 10.478 Å, and *c* = 4.3763 Å^38^. The relaxed lattice constants for monolayer phosphorene are *a* = 3.295 Å, *b* = 4.618 Å which are good agreement with other theoretical calculations^39^. Moreover, in all calculations of the electronic and magnetic structures, the Fermi level is set as the energy zero point. We will hereafter refer to a ZPNR with *n* zigzag chains as an *n*ZPNR. Here, following the conventional notation, “*n*” represents the number of zigzag chains along the width of the nanoribbon.

**Figure 1:**
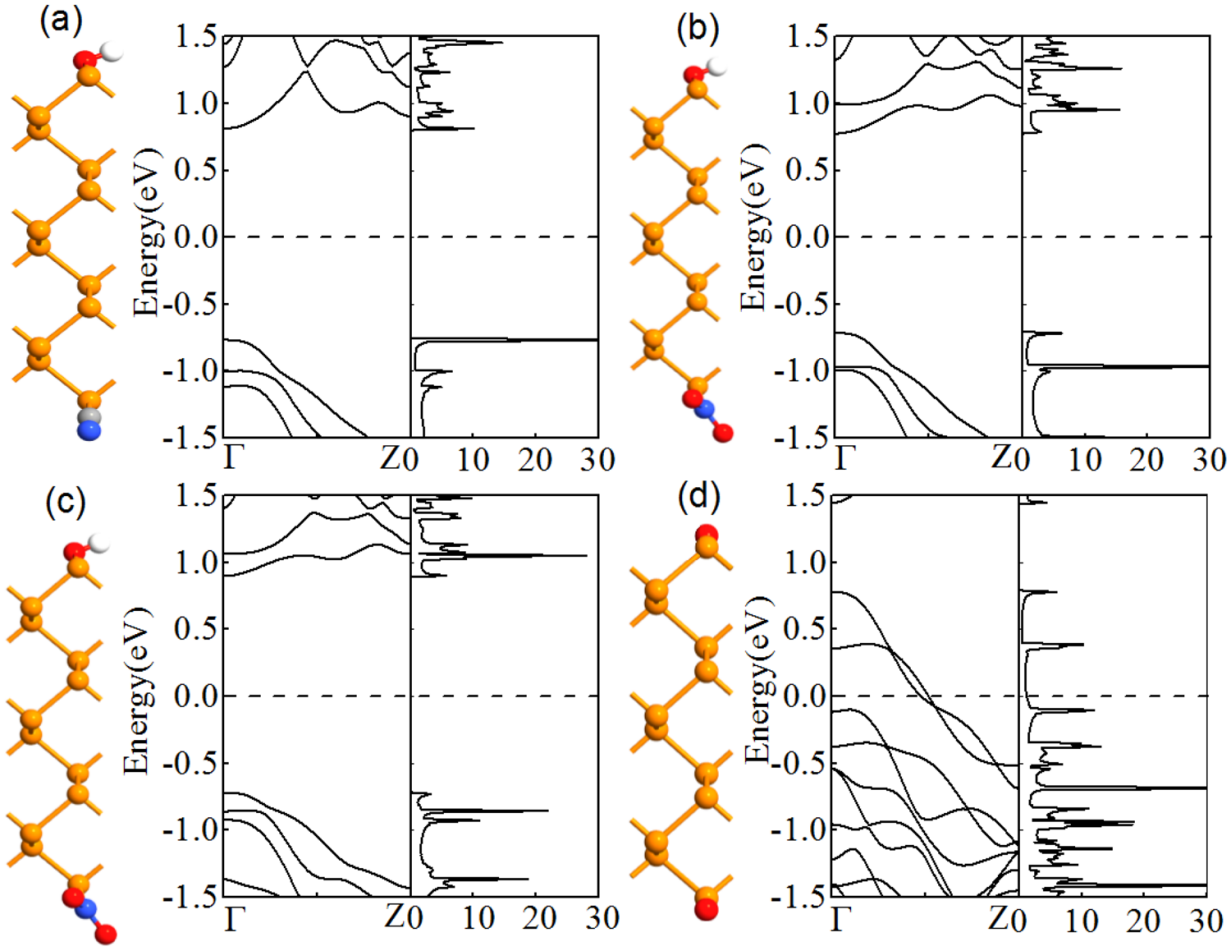
Optimized unit-cell geometries, the band structures (BS) and density of states (DOS) of (**a**) OH-6ZPNR-CN (**b**) OH-6ZPNR-NO_2_ (**c**) NH_2_-6ZPNR-NO_2_ (**d**) 6ZPNR-2O nanosructures. The yellow, red, white, blue and grey colors denote the phosphorene, oxygen, hydrogen, nitrogen and carbon atoms, respectively. The Fermi level is plotted with a black dashed line.

**Figure 2:**
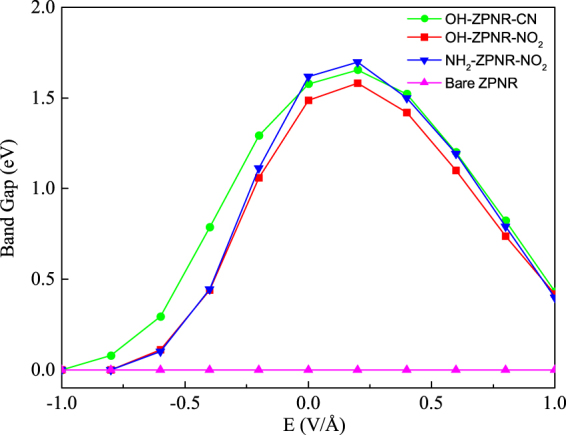
Energy gaps of edge states of the studies nanoribbons versus a transverse electric field.

**Figure 3:**
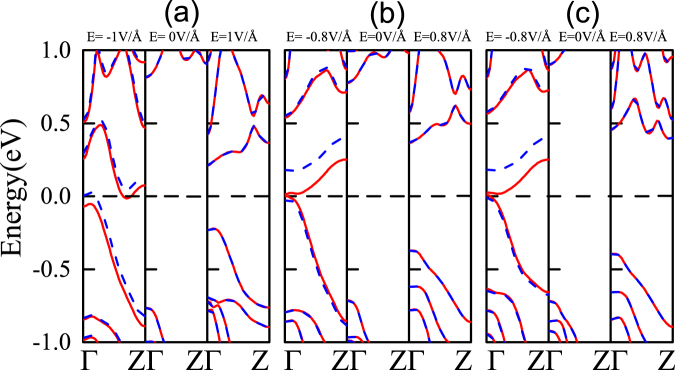
The band structure modifications of (**a**) OH-6ZPNR-CN (**b**) OH-6ZPNR-NO_2_ (**c**) NH_2_-6ZPNR-NO_2_ nanosructures for different electric fields, respectively. The Fermi level is plotted with the black dashed line. The red lines denote spin-up bands, while the blue dashed lines denote spin-down bands, respectively.

**Figure 4:**
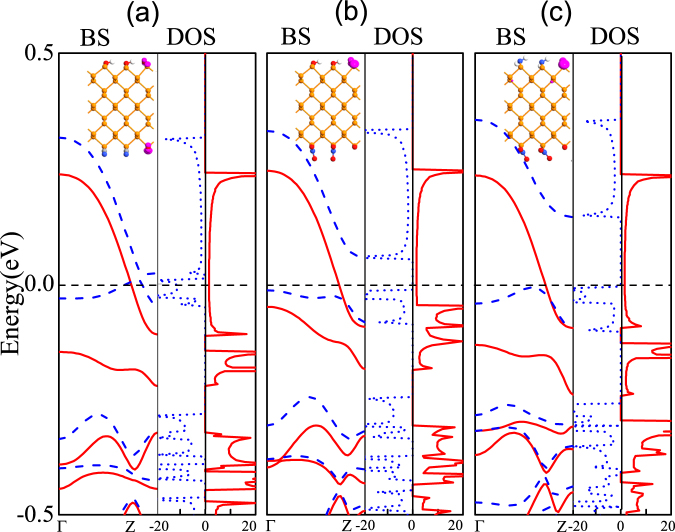
The band structures (BS), the density of states (DOS), and the electron polarization density of oxygen atoms adsorbed on the edge of the black phosphorus in (**a**) OH-6ZPNR-CN-2O, (**b**) OH-6ZPNR-NO_2_-2O (**c**) NH_2_-6ZPNR-NO_2_-2O nanosructures, respectively. The 6ZPNRs with edge functionalization group and edge oxidation at the ratio 2:1. The Fermi level is plotted with the black dashed line. The red lines denote spin-up bands, while the blue dashed lines denote spin-down bands, respectively. The isosurface value is 0.003 |*e*|/*Å*^3^.

To consider different edge-modification effects of 6ZPNRs, four possible structures are designed: (1) OH-ZPNR-CN nanostructure, as manifested in Fig. 1(a), (2) OH-ZPNR-NO_2_ nanostructue, as exhibited in Fig. 1(b), (3) NH_2_-ZPNR-NO_2_ nanostructure, as displayed in Fig. 1(c), and (4), 6ZPNR-2O nanostructure, as displayed in Fig. 1(d). BP is etched along a selective crystallographic orientation (e.g., zigzag) with the scanning probe tip^40^ thus, the functionalization scheme is feasible with etching under a specific chemical environment^41,42^. Decoration with a given group is considered up to half-coverage for each edge because full decoration is less likely. All crystal structures are optimized.

The band structures of the ZPNRs with four different edge groups were calculated. We have known that the pristine ZPNR is a metal. For the three different functionalization groups, the ZPNRs are semiconductors with a direct band gap at Γ, as shown in Fig. 1(a)–(c). The band gap is defined as the energy difference between the conduction band minimum (CMB) and valence band maximum (VBM). For the cases with the edge OH/CN, OH/NO_2_, NH_2_/NO_2_, both the CBM and VBM are contributed by the non-edge P atoms (i.e., intrinsic states) in the nanoribbon and located at Γ, which gives a direct band gap. Edge oxidized ZPNRs show metallic band structures caused by the larger electronegativity of oxygen relative to phosphorene, as shown in Fig. 1(d). In the following, we discuss the effect of external transverse electric field, and the different proportions of functionalization group and edge oxidation on the band structures.

## Results and Discussion

To evaluate the energetically relative stability of the ZPNR with different terminations, the edge formation energy is defined as, *E*_*f*_ = (*E*_*t*_ − *E*_*ribbon*_ − *nE*_1_ − *mE*_2_)/(*n* + *m*), where *E*_*t*_ is the calculated total energy of chemical-functionalized ZPNRs, *E*_*ribbon*_ = −3170.05 eV is the calculated total energy of bared ZPNRs. *E*_1_ and *E*_2_ are respectively the energy of the isolated functional groups, and *n* and *m* are respectively the number of functional groups saturated in per unit cell. The calculated results are shown in Table 1, and it is seen that the binding energy are all negative, which implies that the chemical-termination with OH/CN, OH/NO_2_ NH_2_/NO_2_, and edge oxidation are all exothermic reaction from the bared zigzag edges of black phosphorene. So the OH/CN, OH/NO_2_, NH_2_/NO_2_ -edge saturation and edge oxidation can effectively enhance the stability of black phosphorene nanoribbon.

**Table 1:** Formation energies in different chemical modification.

Structure	E_*t*_(eV)	E_1_(eV)	E_2_(eV)	E_*f*_(eV)
OH-6ZPNR-CN	−4050.10	−447.30	−423.44	−4.66
OH-6ZPNR-NO_2_	−4761.10	−447.30	−1136.93	−3.41
NH_2_-6ZPNR-NO_2_	−4613.86	−300.46	−1136.93	−3.21
ZPNR-2O	−4039.85	−427.81	−427.81	−7.09

Figure 2 illustrates energy gaps versus the external transverse electric field. Since edge functionalization of ZPNRs lack mirror symmetry with respect to their central axis, not only the intensity but also the direction of the field have influence on the electronic properties of the system. Therefore, we consider both opposite directions (positive and negative) electric fields in our calculations. An in-plane homogeneous transverse electric field oriented from top to bottom, is applied to edge-modified ZPNRs and referred to as the positive direction. The upper functionalization groups are OH and NH_2_, while the lower functionalization groups are CN and NO_2_. Our SGGA-PBE results show that the pristine ZPNRs are metals, and the PNRs with the edge phosphorus atoms passivated using OH/CN, OH/NO_2_ and NH_2_/NO_2_ are semiconductors. By applying a positive transverse electric field, the band gap of the edge states increases to a maximum (about 1.7 eV) and then decreases (from *E* = 0.2 V/*Å*) but remains semiconducting in the large range of the external electric field. However, by applying a negative transverse electric field, the band gap of the edge states decreases and even becomes zero, i. e., the metal phase. As the electric field increases, the number of electrons at each edge atom decreases/increases. This gradual decrease/increase of the electron number causes a nonmonotonic variation of the gap between spin-up and spin-down bands near the Fermi level. It is worth mentioning that switching the position of the functional groups to the opposite edges is equivalent to reversing the electric field direction.

Figure 3(a)–(c) summary the results of band structure modifications for the functionalized ZPNRs in the presence of external transverse electric field. The application of the external electric field induces spin polarization. When *E*_*ext*_ = −1.0 V/*Å*, there is small spin polarization in the OH-ZPNR-CN nanostructures, the spin-splitting becomes obvious when *E*_*ext*_ = −0.8 V/*Å* for OH-6ZPNR-NO_2_ and NH_2_-6ZPNR-NO_2_ nanosturctures. Here we elucidate the mechanism responsible for the crossover from nonmagnetic to magnetic phase in ZPNRs. We consider three different combinations of functional groups, OH/CN, OH/NO_2_ and NH_2_/NO_2_, where each configuration consists of an electron donating group at one edge and a withdrawing group at the other edge. The upper edges (OH, NH_2_) and lower edges (CN, NO_2_) of the ZPNRs are oppositely charged and therefore an inherent negative electric field is induced in the system. When applying the positive external electric field, the effect of the applied electric field *E*_*ext*_ and the inherent electric field *E*_*inh*_ induced by the asymmetric functional saturation cancel each other, therefore there is not spins splitting for positive transverse electric field. By flipping the direction of the electric field, the induced potentials at the edges by the applied inherent electric fields are along the same direction. When the electron moves along an electric field **E**, it experiences an effective magnetic field *B*_*eff*_ ~ **E** × ***p***/*mc*^2^ in its rest-frame (where *c* is the speed of light, **p** is the electron momentum)— a field that also induces a momentum-dependent Zeeman energy. When the applied electric field is above the critical value (*E*_*ext*_ = −1.0 V/*Å* in Fig. 3(a), *E*_*ext*_ = −0.8 V/*Å* in Fig. 3(b) and (c)), the perceptible spin splitting appears.

We used oxygen atoms adsorbed on the edges of the black phosphorus nanoribbons, to explore whether the oxygen atom adsorption can make the ZPNRs become magnetic^43^. If the upper and lower layers are labeled by *C*1 and *C*2 respectively, the chemical functional group and the oxygen atom are absorbed at the *C*1 side, sequentially another functional group and oxygen atom are absorbed at the *C*2 side. All structures are optimized. The edge formation energies of the ZPNRs with different edge functionalization groups and edge oxidation to 2:1 ratio are respectively −5.48 eV for edge functionalization group OH/CN, −4.62 eV for OH/NO_2_, and −4.47 eV for NH_2_/NO_2_, which are much lower than that of the ZPNRs only with corresponding edge functionalization groups, indicating edge oxidation can further effectively enhance the stability of ZPNRs with different edge functionalization groups. The electron polarization density, band structures and density of states of the ZPNRs with different edge functionalization groups and edge oxidation to 2:1 ratio were calculated, as shown in Fig. 4. The ZPNRs with edge functionalization group OH/CN and edge oxidation to 2:1 ratio are spin-polarized metal, while the ZPNRs with edge functionalization group OH/NO_2_(NH_2_/NO_2_) and edge oxidation to 2:1 ratio are spin-polarized semimetals even at zero external electric field. The oxygen atoms in non-metallic atoms make edge-functionalized ZPNRs produce significant spin splitting. From the figures of the electron polarization densities and Table 2, one find that the electron polarization density mainly comes from the contributions of edge O atoms as well as their adjacent P atoms. The edge P atoms do not form a saturated bond with the decorated O atoms. The *p*_*z*_-orbitals of the edge O and P atoms form a relatively weak P-O bond in the ribbon plane due to the a minimal overlap of the wavefunction. Therefore, these unsaturated bonds bring magnetic moments in oxygen-saturated ZPNRs. Previous work has confirmed that ZGNRs can display half-metallic properties when *E*_*ext*_ is applied^29^. In contrast, our proposal does not require external electric fields. The inherent electric field *E*_*inh*_ induced by the asymmetric functional saturation makes the charge transfer between two edges, that is, the electron accumulation/depletion at both egdes. There are net spin-up charge polarization density at the top of the ZPNRs.

**Table 2:** Magnetic moment of the oxygen and phosphorus atom at the upper and lower edges.

Structure	Upper Edge		Lower Edge	
	M_*O*_(*μ*_*B*_)	M_*P*_(*μ*_*B*_)	M_*O*_(*μ*_*B*_)	M_*P*_(*μ*_*B*_)
OH-6ZPNR-CN-2O	0.049	0.069	0.097	0.106
OH-6ZPNR-NO_2_-2O	0.105	0.154	0	0.007
NH_2_-6ZPNR-NO_2_-2O	0.157	0.214	0.010	0.010

**Figure 5:**
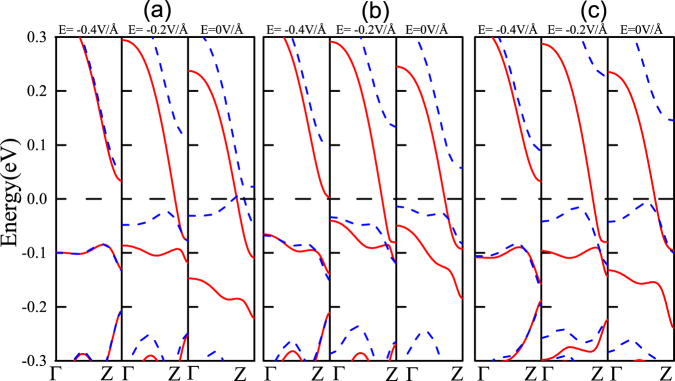
The same as Fig. 4, but for different electric fields.

The effects of edge chemistry on the electronic properties of ZPNRs is expected to alter their response towards the external fields. We apply the transverse electric field along the width of edge-modified ZPNRs. As in Fig. 5(a), the ZPNRs with edge functionalization group OH/CN and edge oxidation to 2:1 ratio in the absence of an external field are found to be metal and present a rich spectrum of electronic characteristics ranging from metallic through half-metallic to semiconducting under the external negative electric field. At the negative direction a lower external field intensity (*E*_*ext*_ = −0.2 V/*Å*) is required to induce half-metallicity, as shown in Fig. 5(a). The ZPNRs with edge functionalization group OH/NO_2_(NH_2_/NO_2_) and edge oxidation to 2:1 ratio are spin-polarized half metals in the absence of an external field. When the applied negative electric field is smaller than 0.2 V/*Å*, half-metallicity is preserved, which are shown in Fig. 5(b) and (c). It shows that the edge functionalization groups OH/NO_2_ and NH_2_/NO_2_ and edge oxidation to 2:1 ratio would be proper structures to obtain half-metallic ZPNRs because they offer a wider electric field range of preserving half-metallicity. As the magnitude of the electric field increases and exceeds the half-metallicity conditions (e. g., *E*_*ext*_ = −0.4 V/*Å*), the ground state of the system will be transformed from the half-metallic nature to the semiconductor. This can be understood easily. The applied external electric field *E*_*ext*_ and inherent electric field induced by the asymmetric functional saturation *E*_*inh*_ produce an electrostatic potential difference between the two edges. The electrostatic potential at the lower and upper edges are thus respectively raised and lowered^44^. The charge in the spin-up conduction band is located at the lower nanoribbon edge when *E*_*ext*_ = 0 V/*Å*. Thus the raising of the electrostatic potential by *E*_*tot*_ shifts spin-up conductance band upwards. When *E*_*ext*_ = −0.4 V/*Å*, there is not energy band pass through the Fermi energy.

**Figure 6:**
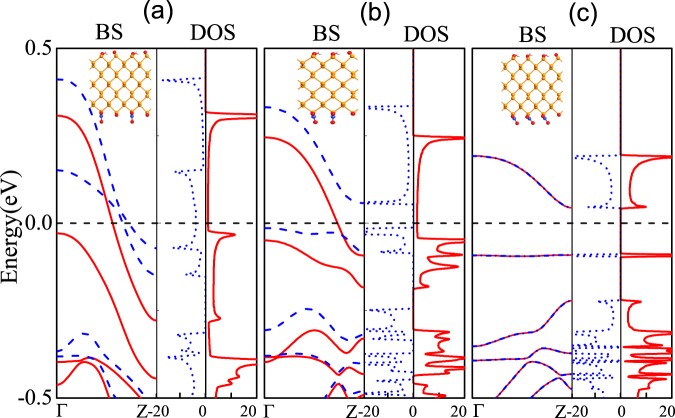
The band structures and the density of states in (**a**) one functional group OH/NO_2_ and one edge oxidation (**b**) two repeating functional group OH/NO_2_ and one edge oxidation (**c**) three repeating functional group OH/NO_2_ and one edge oxidation on the 6ZPNRs, respectively. The Fermi level is plotted with a black dashed line. Red solid and blue dashed lines denote the spin-up and spin-down electron bands, respectively.

It would be interesting to investigate whether edge oxidation may be used to further control the half-metallic behaviour of edge-functionalized ZPNRs. In what follows, we focus the discussion on OH-6ZPNR-NO_2_ nanostructure as a reference. The optimized unit-cell geometries, band structures and density of states of the ZPNRs with edge functionalization group OH/NO_2_ and different proportions edge oxidation were calculated, as shown in Fig. 6. For one functional group OH/NO_2_ and one edge oxidation on the ZPNRs in unit cells, the system is spin-polarized metal, as shown in Fig. 6(a). And for three repeating functional group OH/NO_2_ and one edge oxidation on the ZPNRs in unit cells, the system is semiconductor, as shown in Fig. 6(c). Only when the ZPNRs with two repeating functional group edge oxidation and one edge oxidation in unit cells, the system is half-metal, which is shown in Fig. 6(b). With the decrease of O ratio from 50%, 33.3%, to 25%, the total magnetic moment of the system decreases from 0.771 *μ*_*B*_, 0.440 *μ*_*B*_ to zero. Therefore the band structures can be controlled by the proportion of the functional groups and edge oxidation.

## Conclusion

In summary, based on the first-principles calculation, the electronic structure and magnetic properties of ZPNRs terminated with different edge-functionalized are investigated theoretically. We find that the spin polarization in the ZPNRs can even approach 100%, i. e., the half metal phase. Notice that the band gap of the half metal phase can approach 0.24 eV, which make the half metal phase exist at room temperature. Interesting, the half metal phase can be controlled by the edge functional groups and external electric fields. Our results indicate that edge functionalization of ZPNRs may open the way for the design of new nano-electronic and nano-spintronic devices.
